# Whole genome sequencing of *Mycobacterium bovis* directly from clinical tissue samples without culture

**DOI:** 10.3389/fmicb.2023.1141651

**Published:** 2023-05-18

**Authors:** Mohamed Zeineldin, Patrick Camp, David Farrell, Kimberly Lehman, Tyler Thacker

**Affiliations:** National Veterinary Services Laboratories, Veterinary Services, Animal and Plant Health Inspection Service, United States Department of Agriculture, Ames, IA, United States

**Keywords:** *Mycobacterium bovis*, sequencing, target enrichment, clinical samples, diagnostic

## Abstract

Advancement in next generation sequencing offers the possibility of routine use of whole genome sequencing (WGS) for *Mycobacterium bovis* (*M. bovis*) genomes in clinical reference laboratories. To date, the *M. bovis* genome could only be sequenced if the mycobacteria were cultured from tissue. This requirement for culture has been due to the overwhelmingly large amount of host DNA present when DNA is prepared directly from a granuloma. To overcome this formidable hurdle, we evaluated the usefulness of an RNA-based targeted enrichment method to sequence *M. bovis* DNA directly from tissue samples without culture. Initial spiking experiments for method development were established by spiking DNA extracted from tissue samples with serially diluted *M. bovis* BCG DNA at the following concentration range: 0.1 ng/μl to 0.1 pg/μl (10^–1^ to 10^–4^). Library preparation, hybridization and enrichment was performed using SureSelect custom capture library RNA baits and the SureSelect XT HS2 target enrichment system for Illumina paired-end sequencing. The method validation was then assessed using direct WGS of *M. bovis* DNA extracted from tissue samples from naturally (*n* = 6) and experimentally (*n* = 6) infected animals with variable Ct values. Direct WGS of spiked DNA samples achieved 99.1% mean genome coverage (mean depth of coverage: 108×) and 98.8% mean genome coverage (mean depth of coverage: 26.4×) for tissue samples spiked with BCG DNA at 10^–1^ (mean Ct value: 20.3) and 10^–2^ (mean Ct value: 23.4), respectively. The *M. bovis* genome from the experimentally and naturally infected tissue samples was successfully sequenced with a mean genome coverage of 99.56% and depth of genome coverage ranging from 9.2× to 72.1×. The spoligoyping and *M. bovis* group assignment derived from sequencing DNA directly from the infected tissue samples matched that of the cultured isolates from the same sample. Our results show that direct sequencing of *M. bovis* DNA from tissue samples has the potential to provide accurate sequencing of *M. bovis* genomes significantly faster than WGS from cultures in research and diagnostic settings.

## Introduction

Bovine tuberculosis (bTB), caused by *Mycobacterium bovis*, is widely recognized as a common zoonotic diseases worldwide (Palmer et al., 2012). The potential transmission of *M. bovis* from cattle to humans has prompted many countries to implement national eradication programs to eradicate bTB from cattle and wild animals (Schiller et al., 2010). While many eradication programs have succeeded in reducing the prevalence of bTB in several countries, disease eradication remains elusive in most countries (Quadri et al., 2020).

Traditionally, clinical diagnosis and genotyping of *M. bovis* was most often confirmed by direct culture of *M. bovis* from clinical samples, which is technically laborious and time consuming (Gormley et al., 2014). Whole-genome sequencing (WGS) is a powerful and cost-effective tool that offers unprecedented resolution for outbreak investigations, bacterial genotyping, pathogen evolution, and phylogenetic relationships among members of the same species (Hasman et al., 2014; Salipante et al., 2015; Kohl et al., 2020). As the cost of sequencing has decreased, WGS has become a more routine tool for *M. bovis* genotyping in clinical reference laboratories (Kwong et al., 2015; Orloski et al., 2018). Using whole genome sequences from thousands of *Mycobacterium tuberculosis* complex (MTBC) isolates, the United States Department of Agriculture (USDA) National Veterinary Services Laboratories (NVSL) have developed a high-resolution map of informative single nucleotide polymorphisms (SNPs) that distinguish between different strains and lineages (Orloski et al., 2018). NVSL is able to provide WGS results within the time frame of traditional genotyping (typically within 4–6 weeks from tissue submission), which is then used by federal and state epidemiologists to inform the field investigations during an outbreak. The biggest impediment to rapidly reporting WGS results is waiting for sufficient mycobacteria to grow *in vitro* to enable isolation of sufficient DNA—a process that takes weeks (Cabibbe et al., 2020; Goig et al., 2020). As a result, efforts are being undertaken to develop techniques for WGS of mycobacteria directly from clinical specimens without the need for culture (Brown et al., 2015; Goig et al., 2020; Kayomo et al., 2020). Such methods are challenged by low amounts of mycobacterial DNA in a clinical sample, hardiness of the mycobacterial cell wall, and the high abundance of host and other microbial DNA in clinical samples (Hasman et al., 2014; Brown et al., 2015). In addition, mycobacterial genomes have a substantial number of repetitive elements and a GC content of 65% across the genome, which constitute the main challenges for WGS library preparation and data analysis (Periwal et al., 2015). To overcome these limitations, methods for targeted enrichment of mycobacterial DNA and depletion of host DNA are required before undertaking WGS directly from clinical samples (Votintseva et al., 2017). Recently, a number of commercial DNA enrichment strategies have been developed to sequence the microbial genomes within mixed samples (Clark et al., 2018). Using a combination of target DNA enrichment and WGS to capture all known variations found within a bacterial genome directly from clinical samples reduces the time and cost associated with traditional culture and genotyping, making these technologies an attractive option for reference laboratories (Garcia-Garcia et al., 2016).

One promising approach to target DNA enrichment involves using the SureSelect target enrichment system to selectively capture targeted fragments of genomic DNA (Giuffre et al., 2011). This system uses custom-designed biotinylated RNA probes (baits) that bind to sequences of interest (Giuffre et al., 2011). The biotinylated RNA baits bound to target DNA are concentrated using streptavidin-labeled beads, allowing library preparation and Illumina paired-end sequencing. This method has recently been used to enrich and sequence the genome of a number of microorganisms, including *M. tuberculosis* (Brown et al., 2015; Doyle et al., 2018; Cai et al., 2019); however, currently published methods are limited to isolate genotyping and drug resistance profile prediction (Nimmo et al., 2019; Kizny Gordon et al., 2021).

To date, no study has been reported that directly sequences the whole genome of *M. bovis* DNA from infected tissue samples. The aim of this preliminary study was to assess the diagnostic utility of the SureSelect target enrichment method in sequencing *M. bovis* genomic DNA from spiked samples with BCG to establish limits of detection, from experimentally infected animal tissue to establish assay viability and from naturally infected animal samples to assess real world sample applications and comparison of genomic characterization to traditional culture methods.

## Materials and methods

### BCG spiked tissue samples

The tissue samples for method validation were selected from negative samples submitted for routine testing at the NVSL. All tissue samples and *M. bovis* BCG stock cultures were heat inactivated for 30 min at 105°C before DNA extraction. DNA extraction from tissue samples and *M. bovis* BCG stock was performed using the MagMAX™ Total Nucleic Acid Isolation Kit (96 well plate format, Thermo Fisher Scientific, MA, USA) according to the manufacturer’s instructions. Isolated DNA was quantified using a Qubit^®^ fluorometer (Thermo Fisher Scientific, MA, USA) according to manufacturer’s instructions. Four replicates of spiked samples for method development were established by spiking DNA extracted from tissue samples with serially diluted *M. bovis* BCG DNA in 1× TE buffer to reach the following concentration range: 0.1 ng/μl to 0.1 pg/μl (10^–1^ to 10^–4^). The cycle threshold (Ct) value of spiked tissue-BCG DNA was determined using the IS-1081 real-time PCR assay (Dykema et al., 2016).

### Experimentally infected samples for method validation

The method validation was conducted using six granulomatous tissue samples from cattle experimentally infected with *M. bovis* field strain 10-7428 (Palmer et al., 2021). All tissue samples were heat inactivated for 30 min at 105^°^C before DNA extraction. Genomic DNA was extracted directly from tissue samples using the HostZERO microbial DNA kit (Zymo Research, CA, USA) according to the manufacturer’s instructions. Isolated DNA was quantified using the Qubit dsDNA broad range DNA assay (Thermo Fisher Scientific, MA, USA) according to the manufacturer’s instructions. The Ct values of DNA from the samples were estimated using the IS-1081 real-time quantitative PCR (Dykema et al., 2016).

### Clinical tissue samples for method testing

A total of six *M. bovis*-positive granulomatous tissues from naturally infected animals were selected from samples collected at slaughter as a part of the U.S. Tuberculosis Eradication program abattoir surveillance program. All tissue samples were preserved in sodium borate and shipped to NVSL overnight. The samples were prepared for culture using established procedures and cultured in MGIT liquid media and on modified Middlebrook 7H11 solid media. All tissue samples for molecular analysis were heat inactivated for 30 min at 105^°^C before DNA extraction. Genomic DNA was extracted directly from tissue samples using the HostZERO microbial DNA extraction kit (Zymo Research, CA, USA) according to the manufacturer’s instructions. The HostZERO kit was used to lyse host cells and enzymatically degrade released DNA leaving the bacterial cells intact (Heravi et al., 2020), followed by bacterial lysis and DNA isolation. Purified DNA was quantified using the Qubit dsDNA broad range DNA assay (Thermo Fisher Scientific, MA, USA) according to the manufacturer’s instructions. The quantity (Ct value) of *M. bovis* DNA in infected tissue samples was estimated using the IS-1081-real-time PCR assay (Dykema et al., 2016).

### DNA extraction from *M. bovis* isolates from naturally infected animals

Two loopfuls of *M. bovis* colonies growing on Middlebrook 7H11 plates were transferred into 2-ml screwcap tubes containing 400 μl TE buffer and 0.1 mm glass beads then heat-killed for 30 min at 105°C. Bead beating was carried out at full speed for 2 min to achieve microbial cell disruption. Genomic DNA was extracted using the MagMAX™ Total Nucleic Acid Isolation Kit (96 well plate format, Thermo Fisher Scientific, MA, USA) according to the manufacturer’s instructions. Genomic DNA was quantified using the Qubit dsDNA broad range DNA assay (Thermo Fisher Scientific, MA, USA) according to the manufacturer’s instructions.

#### Whole genome sequencing for *M. bovis* isolates

DNA libraries for WGS of *M. bovis* isolates were prepared using the Nextera^®^ XT library preparation kit (Illumina, Inc., San Diego, CA, USA) according to manufacturer’s instructions. Briefly, quantified DNA was tagmented, normalized with magnetic beads, and PCR amplified using the Illumina Enhanced PCR Mix and Nextera XT dual indexed primers. Amplified DNAs were cleaned, and size selected using a double-sided bead purification procedure. Libraries were pooled evenly, denatured and sequenced from both ends using the MiSeq V2 flowcell 500-cycle (2 × 250 bp) following manufacturer’s guidelines (Illumina, Inc., San Diego, CA, USA).

### SureSelect target enrichment system

#### Custom capture library RNA baits design

The SureSelect custom capture library baits were synthesized and designed by Agilent Technologies (Agilent, Santa Clara, CA, USA). Probes were designed using the *M. bovis* AF2122/97 reference genome (NCBI RefSeq accession NC_002945.4). Overall, 36,250 RNA probes were designed to cover the entire *M. bovis* genome with 1X tiling. Each probe consisted of 120 nt cRNA, and the total probe size was 4.35Mbp (Supplementary Table 1). The probes were additionally boosted using SureDesign (Agilent, Santa Clara, CA, USA) based on the level of GC content for each probe sequence to allow equal representation of regions that have extreme GC-levels, which are traditionally harder to capture. The specificity of baits was verified by a BLASTn search against the *M. bovis* AF2122 reference genome.

#### Library preparation, target DNA enrichment, and whole genome sequencing

Library preparation, hybridization and enrichment for spiked DNA and DNA extracted from naturally and experimentally infected samples was performed using the SureSelect XT HS2 target enrichment system for Illumina paired-end sequencing libraries (Agilent, Santa Clara, CA, USA) according to manufacturer’s instructions. Briefly, a total of 200 ng input DNA per sample were sheared using an M220 Focused-ultrasonicator (Covaris, Woburn, MA, USA) with the following settings: temperature 20^°^C, duty factor 20%, peak incident power 20 W, and burst rate 200 cpb for 150 s. The fragmented DNA was used for end-repair and adapter ligation. Adapter-ligated libraries were amplified by PCR using SureSelect XT HS2 pre-capture index primer pairs, Herculase II Fusing DNA polymerase and 5x Herculase II buffer with dNTPs. The cycling conditions were as follows: 98°C for 2 min, followed by 12 cycles of 98°C for 30 s, 60°C for 30 s, and 72°C for 1 min; and a final extension at 72°C for 5 min. The PCR products were purified using AMPure XP beads (Agilent, Santa Clara, CA, USA). Quality and quantity of purified pre-capture libraries were determined by TapeStation using a D1000 ScreenTape (Agilent, Santa Clara, CA, USA). Next, 1,000 ng of each library were hybridized to the *M. bovis*-specific RNA capture baits using 60 cycles of incubation at 65°C for 1 min and 37^°^C for 3 s. The hybridized libraries were purified with SureSelect streptavidin magnetic beads (Agilent, Santa Clara, CA, USA). The beads with captured DNA were then washed one time with SureSelect wash buffer 1 and six times with pre warmed SureSelect wash buffer 2 to remove non-specific binding. After all wash steps, the beads were suspended in 25 μl of nuclease free water. The captured DNA libraries, bound to streptavidin beads, were amplified by PCR using SureSelect XT HS2 post capture primer mix, Herculase II Fusing DNA polymerase and 5× Herculase II buffer with dNTPs. The cycling conditions were as follows: 98°C for 2 min; followed by 22 cycles of 98°C for 30 s, 60°C for 30 s, and 72°C for 1 min; and a final extension at 72°C for 5 min. After PCR amplification, streptavidin beads were removed, and the amplified PCR products were further purified with AMPure XP beads (Agilent, Santa Clara, CA, USA) and finally eluted using 25 μl of low TE buffer. Final libraries were assessed using an Agilent TapeStation using high sensitivity D 1000 ScreenTape and then pooled for Illumina sequencing. Sequencing of SureSelect enriched libraries was performed using Illumina MiSeq platform using v2 300-cycle cartridges (2 × 150 bp) following manufacturer’s guidelines (Illumina, Inc., San Diego, CA, USA).

#### Illumina sequencing without target enrichment

To compare the effect of target enrichment on DNA sequencing from tissue, spiked *M. bovis*-tissue DNA samples from the spiking experiment underwent library preparation without enrichment using the Nextera^®^ XT library preparation kit (Illumina, Inc., San Diego CA, USA) following manufacturer’s guidelines. The resulting DNA libraries were pooled and sequenced from both ends using Illumina MiSeq using V2 300-cycle (2 × 150 bp) following manufacturer’s guidelines (Illumina, Inc., San Diego, CA, United States).

#### Bioinformatics analysis, genome alignment and SNP calling

Raw sequence data files were de-multiplexed and converted to fastQ files using Casava v.1.8.2 (Illumina, Inc., San Diego, CA, United States). Sequence read quality was assessed using FastQC software (Andrews, 2017). The demultiplexed fastQ raw files from enriched samples were pre-processed to remove sequencing adaptors and extract the molecular barcode (MBC) sequences using the Agilent Genomics NextGen Toolkit (AGeNT). All pre-processed fastQ files from all samples were then analyzed using validate SNP (vSNP) tool of the US Department of Agriculture-Veterinary Services.^1^ The vSNP tool involves a two-step process to map the sequence reads against the reference genome, determine SNP positions, and compare the called SNPs among related isolates (Duffy et al., 2020). Briefly, the trimmed sequence reads were mapped against the reference genome *M. bovis* AF2122/97 (NCBI RefSeq accession NC_002945.4) with Burrows Wheeler Aligner (Andrews, 2017) and Samtools (Andrews, 2017). All PCR duplicates were marked and removed from the alignment files using Picard v.2.10.5.^2^ SNPs with a quality score >300 and AC = 2 were called using FreeBayes^3^ and visually validated with Integrative Genomics Viewer (IGV) (Robinson et al., 2011). SNP groups for closely related isolates were determined and labeled based on the defining SNP’s position with respect to the reference genome. Maximum likelihood phylogenetic trees were constructed based on the output SNP alignment using RAxML software (version 8.2; GTRCATI model). The resulting phylogenetic tree was visualized using FigTree v1.4.3.^4^ Spoligotypes were called using the vSNP pipeline with the “spoligo” function, which outputs a text file that lists the read counts for each of the spacer regions. SB codes are obtained by cross-referencing the calculated spoligo against the *M. bovis* Spoligotype Database.^5^ Sequenced reads were classified using Kraken2 against Kraken database previously constructed from all available RefSeq genomes for bacteria, archaea, viruses, protozoa, and fungi (Wood et al., 2019).

All non-*M. bovis* reads identified by Kraken were removed before assembly. Draft genome assemblies for sequenced samples were conducted using SPAdes (Bankevich et al., 2012). Finally, draft genome comparison from *M. bovis* sequenced directly from clinical samples and from cultured isolates was carried out using progressive Mauve alignment using default parameters (Darling et al., 2010).

### Data availability

All sequence reads generated from this study were submitted to the sequence read archive on the NCBI website under a bio-project accession number PRJNA752391.

## Results

### Direct sequencing of spiked DNA samples

Our initial experiments focused on method development and optimization using spiked DNA samples to identify the proportion of *M. bovis* DNA that could be successfully sequenced directly from tissue samples. Across all spiked DNA samples, direct WGS generated a total of 58,977,030 raw sequence reads (mean number of sequences per sample: 3,686,064.37; median: 3,749,952; range: 1,012,682–5,817,796) with an average read quality score of 36.6. The results of the spiked sample sequencing are shown in Table 1.

**TABLE 1:** Direct sequencing of DNA from tissue samples spiked with serially diluted *M. bovis* BCG DNA at different concentration range.

Spiked sample ID	BCG DNA dilution	CT value	*M. bovis* read (%)	Bos taurus reads (%)	Mean read quality	Total reads	All mapped reads	Reference with coverage (%)	Average depth of coverage	Quality SNP count	Group placements	Bovis SB code
**Sample1**	10^–1^	21.1	67	31	36.8	5,817,796	2,521,642	98.96	78.2×	811	Mbovis-21ABCG	SB0120
**Sample2**	10^–1^	19.5	97	2	36.3	4,974,906	4,346,011	98.97	135.2×	823	Mbovis-21ABCG	SB0120
**Sample3**	10^–1^	20.4	87	11	36.3	3,413,870	2,073,719	98.97	80.9×	808	Mbovis-21ABCG	SB0120
**Sample4**	10^–1^	20.2	98	2	35.2	5,303,126	4,279,021	99.00	140.6×	923	Mbovis-21ABCG	SB0120
**Sample5**	10^–2^	23.4	46	48	37.0	4,492,766	714,204	98.88	22.0×	762	Mbovis-21ABCG	SB0120
**Sample6**	10^–2^	23.7	68	21	35.4	3,299,936	526,741	98.78	16.4×	695	Mbovis-21ABCG	SB0120
**Sample7**	10^–2^	22.6	78	18	36.7	4,576,668	1,694,642	99.12	52.7×	809	Mbovis-21ABCG	SB0120
**Sample8**	10^–2^	24	59	33	36.3	5,434,196	441,490	98.67	13.7×	661	Mbovis-21ABCG	SB0120
**Sample9**	10^–3^	28.9	4	88	37.6	1,028,550	36,866	46.62	1.0×	28	No defining SNP	Not defined
**Sample10**	10^–3^	26.9	5	83	36.9	3,747,062	72,414	68.68	2.0×	59	No defining SNP	Not defined
**Sample11**	10^–3^	27.3	4	83	36.4	2,015,436	46,371	60.97	1.4×	21	No defining SNP	Not defined
**Sample12**	10^–3^	28.8	4	75	35.8	3,590,974	48,045	59.72	1.3×	23	No defining SNP	Not defined
**Sample13**	10^–4^	30.3	1	92	37.7	1,012,682	12,763	13.81	0.2×	5	No defining SNP	Not defined
**Sample14**	10^–4^	29.6	1	90	37.2	3,752,842	17,015	17.36	0.3×	26	No defining SNP	Not defined
**Sample15**	10^–4^	30.8	1	87	36.3	4,916,224	14,251	9.33	0.2×	30	No defining SNP	Not defined
**Sample16**	10^–4^	30.7	1	88	37.7	1,599,996	19,906	21.28	0.4×	7	No defining SNP	Not defined

All sequence reads were aligned against the *M. bovis* AF2122/97 (NCBI RefSeq accession NC_002945.4) reference genome using the vSNP tool. Our analysis revealed that the genome coverage, depth of coverage, and the ratio of host reads to *M. bovis* reads generated per sample was linear (Supplementary Figure 1). Direct sequencing of spiked DNA samples achieved 99.1% mean genome coverage (mean depth of coverage: 108×) and 98.8% mean genome coverage (mean depth of coverage: 26.4×) for tissue DNA spiked with BCG DNA at 10^–1^ (mean Ct: 20.3) and 10^–2^ (mean Ct: 23.4), respectively. Tissue DNA spiked with BCG DNA at 10^–3^ (mean Ct: 27.9) and 10^–4^ (mean Ct: 30.3) achieved only 58.9% mean genome coverage (mean depth of coverage: at 1.4×) and 15.4% mean genome coverage (mean depth of coverage: at 0.2×), respectively. Tissue DNA spiked with BCG DNA at 10^–1^ and 10^–2^ showed 46–98% of the resulting sequenced reads assigned as *M. bovis* reads (Table 1), while samples spiked with BCG DNA at 10^–3^ and 10^–4^ showed 75–92% of the resulting sequenced reads assigned as *Bos taurus* (Table 1).

DNA samples spiked with BCG DNA at 10^–1^ and 10^–2^ were correctly assigned to *M. bovis*-21A, the group that contains BCG, with an average of 786.5 quality SNPs(range of 661–923) per genome (Table 1). Final SNP alignments for each sequenced *M. bovis* genome from spiked samples compared to other *M. bovis* BCG genomes from the NVSL *M. bovis* genome database is shown in Supplementary Table 2. The results of this spiking experiment suggest that samples with Ct values of <26 contain enough *M. bovis* to directly sequence and genotype from the tissue; however, samples with Ct values >26 do not have sufficient *M. bovis* DNA to directly sequence from the tissue.

The efficiency of the SureSelect target enrichment technique was validated by sequencing DNA from tissue samples spiked with serially diluted *M. bovis BCG* DNA at different concentrations, 10^–1^ to 10^–4^, without target enrichment using the Nextera^®^ XT Illumina library preparation kit and analyzed using our current WGS workflow. Without the SureSelect target enrichment, the spiked DNA samples with BCG DNA at 10^–1^ had a genome coverage of 0.8% with 0× depth of coverage (Supplementary Table 3). The results of the spiking experiment suggest that our target enrichment method significantly increases the *M. bovis* to host DNA ratio and can effectively capture *M. bovis* DNA from tissue samples.

### Direct sequencing of DNA from tissue samples from experimentally infected animals

The established SureSelect target enrichment and sequencing methods were further tested using six granulomatous tissue samples from experimentally infected animals. All samples tested positive for *M. bovis* using IS-1081 real-time PCR with Ct values that ranged from 23.4 to 27.4 cycles (Table 2). All sequenced samples met the quality criteria for downstream analyses with an average read quality score of 37.4. The resultant sequence reads were mapped against the *M. bovis* AF2122/97 reference genome using vSNP. The *M. bovis* genomes from all experimentally infected tissue samples were successfully sequenced with a mean genome coverage of 99.42% and mean depth of genome coverage ranging from 9.2× to 72.1×. Kraken analysis showed that the ratio of *M. bovis* to *B. taurus* reads was variable between samples, with samples that had lower Ct values providing higher *M. bovis* reads and less *B. taurus* reads (Supplementary Figure 2).

**TABLE 2:** Sequencing results of *M. bovis* DNA directly sequenced from tissue samples from experimentally infected animals.

Sample ID	CT value	Mean read quality	Total reads	All mapped reads	Unmapped reads	Contigs	GC content	Genome length	Reference with coverage (%)	Average depth of coverage	Quality SNP count	Group placements	Bovis SB code
Exprimental1	26.9	37.8	3,418,440	620,131	2,099,660	247	65.51	4,231,749	99.62	19.4×	510	Mbovis-07B1	SB2011
Exprimental2	27.4	37.9	3,568,962	300,091	2,683,128	1,109	65.37	3,719,687	98.68	9.2×	391	Mbovis-07B1	SB2011
Exprimental3	23.4	37.3	4,142,574	879,953	1,450,462	217	65.36	4,158,446	99.31	28.0×	512	Mbovis-07B1	SB2011
Exprimental4	23.6	37.3	5,080,070	1,688,629	1,720,756	156	65.46	4,234,246	99.60	53.7×	548	Mbovis-07B1	SB2011
Exprimental5	23.6	37.1	4,390,444	2,277,800	731,104	124	65.57	4,279,781	99.68	72.1×	551	Mbovis-07B1	SB2011
Exprimental6	26.4	37.2	5,218,554	711,299	2,614,880	242	65.51	4,231,654	99.61	22.0×	547	Mbovis-07B1	SB2011
*M. bovis* 10-7428	11	32.5	1,526,346	1,534,190	2,032	116	65.54	4,217,396	99.53	97.3×	540	Mbovis-07B1	SB2011

Single nucleotide polymorphism based on the alignment to *M. bovis* AF2122/97 reference genome were called, and all genomes clustered within Mbovis-07B1 group with an average of 514.1 quality SNP (range of 391–551) per genome (Table 2). The direct sequenced genomes from experimentally infected animals fell in clade NC_002945.4:1668725 with no SNP difference from *M. bovis* field strain 10-7428 that was used for the experimental infection. Only one sample (experimental2) showed 5 SNPs difference from *M. bovis* 10-7428, and this was due to the low sequence coverage of this sample. Final SNP alignments for each sequenced *M. bovis* genome from experimentally infected animals compared to *M. bovis* genomes within Mbovis-07B1 group are shown in Supplementary Table 4. Finally, the spoligotyping matched between the sequenced genomes from tissue samples from experimentally infected animals and *M. bovis* field strain 10-7428 which was used for the experimental infection (Table 2).

### Direct sequencing of DNA from tissue samples from naturally infected animals

The SureSelect target enrichment technique was then applied to sequence DNA extracted from six clinical granulomatous tissue samples from naturally infected animals. All lesions from naturally infected animals were evaluated by a Pathologist. All tissue samples were histologically consistent with tuberculosis and acid-fast bacteria were detected. In additional to histology, all lesions were evaluated using the IS-1081 real-time quantitative PCR and shown to contain mycobacteria from the Mycobacterium tuberculosis complex with Ct values that ranged from 20.2 to 29.5 cycles (Table 3). All of the bait-enriched and sequenced samples met the quality criteria for downstream analyses with an average read quality score of 36.2 and an average GC content of 65.4. Across all DNA samples sequenced directly from tissue, WGS generated a total of 37,312,362 raw sequence reads (mean number of sequences per sample: 6,218,727; median: 5,474,791; range: 3,482,760–10,470,414) as described in Table 3. The average assembly length of *M. bovis* genome sequenced directly from tissue samples without culture, following the removal of contaminant contigs, was 4,136,746 bp, consistent with the expected size of the *M. bovis* genome (Table 3).

**TABLE 3:** Sequencing results of *M. bovis* DNA directly sequenced from tissue samples from naturally infected cattle and the genomes of corresponding isolates cultured from the same sample.

Sample ID	Ct value	Total reads	Mean read quality	All mapped reads	Unmapped reads	Contigs	GC content	Genome length (bp)	Reference with coverage	Average depth of coverage	Quality SNP count	Group placements	Bovis SB code
21-3386_tissue	26	3482760	36.2	861,983	785,816	249	65.29	4,163,606	99.80%	27.6×	342	Mbovis-24	SB0673
21-3386_isolate	10.9	3228036	33.5	3,208,029	1,966	110	65.49	4,266,859	99.73%	170.1×	395	Mbovis-24	SB0673
21-1214_tissue	20.2	4223420	36.6	817,849	781,160	607	65.25	4,063,072	99.89%	26.9×	351	Mbovis-17B1	SB0971
21-1214_isolate	11.1	2134934	33.7	2,125,870	2,452	119	65.48	4,262,402	99.77%	110.1×	355	Mbovis-17B1	SB0971
21-3867_tissue	29.5	8186186	36.5	329,276	1,313,794	707	65.3	3,968,294	99.71%	9.8×	684	Mbovis-17B1	SB0971
21-3867_isolate	11.8	976348	30.5	1,113,963	814	251	65.48	4,205,897	99.79%	48.4×	347	Mbovis-17B1	SB0971
21-4307_tissue	29.3	10470414	36.1	302,762	21,144	1,049	65.5	3,743,070	99.01%	9.2×	385	Mbovis-16C	SB0673
21-4307_isolate	11.3	1345916	34.0	1,338,356	8,690	123	65.46	4,256,741	99.59%	69.2×	359	Mbovis-16C	SB0673
21-6312_tissue	28.4	5076326	37.7	634,331	3,088,980	215	65.51	4,223,426	99.63%	19.2×	685	Mbovis-06A	SB0145
21-6312_isolate	11	1448764	31.6	1,451,613	2,936	113	65.44	4,216,956	99.33%	77.8×	549	Mbovis-06A	SB0145
21-6508_tissue	29	5873256	37.6	356,850	4,539,502	772	65.6	4,028,519	99.34%	10.9×	511	Mbovis-07B	SB0327
21-6508_isolate	10.9	2,817,170	32.1	2,780,522	4,372	109	65.47	4,242,117	99.53%	141.8×	561	Mbovis-07B	SB0327

The *M. bovis* genome from all naturally infected tissue samples was successfully sequenced with mean genome coverage of 99.56% and mean depth of genome coverage ranging from 9.2× to 27.6×. Kraken sequence read classification analysis showed that samples with lower Ct values had a higher percentage of *M. bovis* reads and lower percentage of *B. taurus* reads (Supplementary Figure 3). Using vSNP, the SNPs called and all isolates were clustered within specific *M. bovis* groups (Table 3). We then compared the genomes sequenced directly from tissue samples to other available *M. bovis* genomes in the NVSL database using SNP data alignment output. Final alignments (SNP matrices) for each sample are shown in Supplementary Tables 5–9. The SNP alignment table for each genome sequenced directly from tissue samples clearly shows that each sequenced genome fell in its defined *M. bovis* group. On average, the genomes sequenced directly from tissue samples were within 15 SNPs from the most recent common ancestor within its defined *M. bovis* group (Supplementary Figure 4). The result of this experiment showed that direct sequencing of DNA using both the SureSelect target enrichment and HostZERO microbial DNA kit to deplete host DNA during the extraction process generates samples with Ct values of <29.5 containing enough *M. bovis* DNA to produce acceptable genome coverage with sufficient depth of coverage to call the defined SNP.

### Congruence between *M. bovis* genomes sequenced from matched pairs of cultured isolate and clinical tissue sample

**FIGURE 1:**
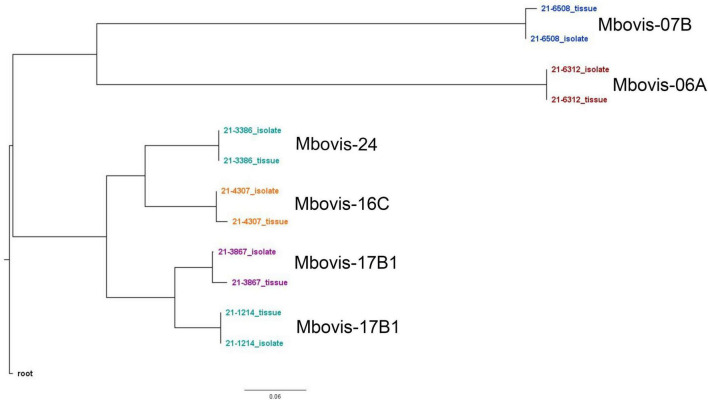
Whole genome SNP-based maximum-likelihood phylogenetic tree of the *M. bovis* genomes sequenced directly from DNA from tissue samples from naturally infected animals and the genomes of the corresponding cultured isolates.

To assess the accuracy of the SureSelect target enrichment and sequencing method, side-by-side comparisons between the genomes sequenced from DNA extracted directly from tissue samples from naturally infected cattle and the genomes of corresponding isolates cultured from the same sample were performed. Whole-genome sequencing of the cultured isolates confirmed the results from the SureSelect target enrichment technique. All spoligotyping information and *M. bovis* group assignments matched between the paired genomes; however, there were some SNP inconsistencies amongst the genomes sequenced directly from clinical samples (Table 3). The genomes sequenced from cultured *M. bovis* showed high depths of coverage compared to genomes sequenced directly from tissue samples (Table 3). The genomes sequenced from cultured *M. bovis* isolates also showed fewer contigs than those from clinical tissue samples, which suggests other DNA sequences were included in the genomic data among the genomes from tissue samples (Table 3). Multiple genome alignments of the *M. bovis* genomes sequenced directly from tissue samples and the genomes of corresponding cultured isolates showed no unique consensus sequence variation between the paired genomes (Supplementary Figure 5). SNP-based phylogenetic trees were also constructed for *M. bovis* genomes obtained using direct sequencing and cultured isolate sequencing based on SNP data alignment output from vSNP (Supplementary Table 10). Even though few SNP differences were observed, almost perfect phylogenetic matches between WGS data obtained from the cultured isolates and directly from tissue samples were observed (Figure 1).

## Discussion

Bovine tuberculosis (bTB), caused by *M. bovis*, is one of the most common causes of zoonotic tuberculosis and is widely recognized as a potential threat to public health (Duffy et al., 2020). Recently, the use of WGS on cultured isolates from granulomatous tissue samples has been shown to be a valuable tool in bTB eradication programs by providing faster option for strain classification and tracing infectious sources (Trewby et al., 2016; Crispell et al., 2017). However, *M. bovis* culture prior to WGS is laborious and time consuming; and for the submitters waiting for the results, this can be a frustrating wait (Orloski et al., 2018). Direct WGS of *M. bovis* DNA from clinical samples without culture may offer a suitable real-time alternative for *M. bovis* genome sequencing which can be utilized to provide valuable information regarding transmission events and to inform contact tracing (Lee and Pai, 2017). However, current direct sequencing techniques are insufficient for recovery of *M. bovis*-specific sequences from tissue samples with overwhelming quantities of host DNA relative to microbial DNA (Votintseva et al., 2017). Recently, several studies have used target enrichment strategies to increase the number of target reads via probe-based enrichment followed by sequencing of the enriched genomic regions (Mamanova et al., 2010; Bodi et al., 2013; Ballester et al., 2016). Previously, SureSelect target enrichment was used to successfully sequence the *M. tuberculosis* genome, define drug resistance profile, and show within-sample diversity directly from sputum samples (Giuffre et al., 2011; Nimmo et al., 2019).

In this study, we have demonstrated, for the first time to our knowledge, the ability to enrich and sequence *M. bovis* DNA directly from granulomatous tissue samples using SureSelect target enrichment and Illumina sequencing. Our workflow enables direct *M. bovis* DNA sequencing and complete downstream genomic analysis, including *M. bovis* group assignment into broader *M. bovis* phylogenetic groups which will aid disease outbreak investigations, to be done in four days from clinical tissue samples, with a comparable cost to WGS workflow of cultured isolates. However, there were some SNP inconsistencies in directly sequenced genomes compared to the genomes sequenced from traditional culture, which suggest some further validating studies before definite conclusion can be made regarding fine scale epidemiological inferences derived from direct sequencing of DNA from tissue samples. Our initial spiking experiment demonstrated a proof-of-principle for sequencing of *M. bovis* genomes directly from clinical samples. The technique was tested using spiked DNA samples with BCG DNA at 10^–1^ (mean Ct: 20.3) and 10^–2^ (mean Ct: 23.4) achieving 99% coverage of the reference genome with sufficient mean depth coverage to perform phylogenomic analyses and assign the samples to the previously defined *M. bovis* group using a set of diagnostic SNPs. In line with other studies, higher mean depth of genome coverage appeared to reflect initial sample microbial DNA. The sequence data generated from the spiking experiment provided an opportunity to compare *M. bovis* genomes sequenced directly without culture with other *M. bovis* BCG genomes from the NVSL database and assign the sequenced genomes to Mbovis-21ABCG group. From the preliminary results of the spiking experiment, we conclude that samples with Ct values of >26 contain few *M. bovis* and would, therefore, be unlikely to produce acceptable genomic data. Conversely, using samples with Ct values of <26 results in a greater proportion of on-target reads and yields genomes with acceptable genome coverage acceptable for further downstream analysis.

When initially planning this experiment, we considered that the overwhelming quantities of host DNA relative to microbial DNA would be the principal technical challenge to be overcome. Although the DNA samples spiked with BCG DNA at 10^–1^ and 10^–2^ tested contained *Bos taurus* DNA, sufficient *M. bovis* DNA was present for successful genome sequencing without specific host DNA depletion. The presence of large amounts of host DNA present in samples spiked with BCG DNA at 10^–3^ and 10^–4^ resulted in unacceptable genome coverage and failed to call the defining SNP. We hypothesized that selectively depleting host DNA in clinical samples during the DNA extraction process would improve the performance of target enrichment and allow us to sequence low titer samples. A previous study showed that the HostZERO DNA extraction kit was successful in reducing host DNA and increased bacterial DNA percentage more than ten-fold (Heravi et al., 2020). The SureSelect target enrichment method was then applied successfully to sequence extracted *M. bovis* DNA, using the HostZERO DNA extraction method, from tissue samples from experimentally (*n* = 6) and naturally (*n* = 6) infected animals. Our results showed that the HostZERO DNA extraction method effectively depletes non-target DNA, yielding a high-quality DNA suitable to sequence the low to mid-titer samples. The *M. bovis* genomes from all experimentally infected and naturally infected tissue samples were successfully sequenced with >99% coverage of the reference genome and sufficient mean depth of coverage to perform phylogenomic analyses and assign the samples to previously defined *M. bovis* groups using a set of diagnostic SNPs. Additionally, using this approach significantly improved the direct sequencing process of lower titer samples while maintaining the ability to accurately detect genome diversity. This is illustrated by the *M. bovis* genome of the lowest titer sample (equivalent to 29.5 Ct using IS-1081 qPCR) being easily sequenced with genome coverage of 99.71% and 9.8× depth of genome coverage. Therefore, samples with a Ct value of 29.5 or less are considered suitable for direct sequencing of *M. bovis* DNA using SureSelect target enrichment technique.

To assess the accuracy of this enrichment and sequencing technique, several comparisons between the *M. bovis* genomes sequenced directly from tissue samples and the genomes of corresponding isolates (i.e., from the same sample) were performed. All sequenced genome pairs were highly similar, however, there were some SNP inconsistencies amongst the paired genomes. Almost all of these are probably due to PCR error, low or no coverage at those SNP location and/or mis-assembly because of host read contamination. In line with other studies, the genomes sequenced from cultured isolate DNA showed higher depth of coverage and contained fewer contigs than those from clinical samples (Doyle et al., 2018; Nimmo et al., 2019). The higher contig numbers among the genomes sequenced directly form tissue samples suggests host sequences or other DNA contamination. Importantly, the RNA probe design of this capture method ensures retention of genome diversity among sequenced genomes. This is illustrated by the phylogenetic analysis of different *M. bovis* groups that clustered separately from one another (Figure 1). Additionally, sequencing the same sample from experimentally infected animals and the spiking experiment at different titers showed reproducible results and similar samples were clustered together.

Besides the capability to sequence the *M. bovis* genome from the clinical tissue samples without culture in only four days, the total cost was also comparable. In this study, it cost US$340 per sample to obtain the WGS directly from clinical tissue samples without culture, which included $30 for SureSelect library preparation, $60 for RNA bait, and $250 per sample (6–8 samples per flow cell) for sequencing.

While our results indicate that the SureSelect target enrichment method is an applicable method for *M. bovis* whole genome sequencing from infected tissue samples within a relatively short time frame, the study exposed limitations that should be considered. A key disadvantage of using the SureSelectXT target enrichment system routinely would be the high cost incurred for WGS of clinical samples in high bTB-burden countries. However, analysis of specific samples of interest would be practicable and could provide important information to a field investigation in a timely matter to support a bTB surveillance or eradication program. A further limitation of this study is that the SureSelect library preparation protocol for direct sequencing includes more pre- and post-PCR cycles than that used for sequencing of cultured isolates, which may increase the risk of sequencing error. To overcome the risk of potential sequencing errors in this study, a stringent approach to data interpretation was adopted. We used high read and mapping quality thresholds that required >98% of the reference genome to be covered by the sequenced reads.

## Conclusion

In conclusion, the findings of our study indicate that performing WGS directly from clinical samples using the SureSelect target enrichment method could be integrated as part of a routine diagnostic procedure and ultimately make real-time WGS of *M. bovis* a reality. This is particularly exciting in view of the importance of bTB to the global cattle industry, and with regard to the potential for developing strategies that could help improve existing eradication programs. Studies on larger numbers of samples are needed to validate this method in clinical samples with low microbial load as well as assess its performance on different specimen types. These studies would help to establish best practice which are most likely to yield high quality genomes of maximum utility in an epidemiological investigation.

## Data availability statement

The datasets presented in this study can be found in online repositories. The names of the repository/repositories and accession number(s) can be found below: https://www.ncbi.nlm.nih.gov/, PRJNA752391.

## Ethics statement

This animal study was reviewed and approved by USDA-National Animal Disease Center Animal Care and Use Committee.

## Author contributions

TT, KL, and MZ designed the experiment. MZ, PC, DF, and KL conducted the experiment. MZ, PC, and DF carried out the laboratory analyses. MZ and TT conducted the data analysis. MZ wrote the manuscript. All authors edited and approved the manuscript for submission.

## Footnotes

1
https://github.com/USDA-VS/vSNP

2
http://broadinstitute.github.io/picard

3
http://bioinformatics.bc.edu/marthlab/FreeBayes

4
http://tree.bio.ed.ac.uk/software/figtree/

5
www.mbovis.org

## Supplementary material

Supplementary Figure 1 Comparison of the percentages of reference genomes coverage, depth of coverage, *M. bovis* reads, host reads on the *y* axis by spiked DNA concertation on the *x* axis for each individual sample. Line of fit shows a linear regression with confidence intervals.

Supplementary Figure 2 Krona charts showing Kraken sequence reads classification and distribution from genomes sequenced directly from tissue samples from experimentally infected animals. Krona plots display the relative abundance and separate taxonomic domains using a radial space-filling display. Taxonomic domains are colored and arranged from the top level of the hierarchy at the center and progressing outward.

Supplementary Figure 3 Krona charts showing Kraken sequence reads classification and distribution from genomes sequenced directly from tissue samples from naturally infected animals. Krona plots display the relative abundance and separate taxonomic domains using a radial space-filling display. Taxonomic domains are colored and arranged from the top level of the hierarchy at the center and progressing outward.

Supplementary Figure 4 Maximum-likelihood phylogenetic tree of the *M. bovis* genomes sequenced directly from DNA from tissue samples from naturally infected animals with other *M. bovis* genomes from NVSL genomes database.

Supplementary Figure 5 Mauve multiple genome alignments of the *M. bovis* genomes sequenced directly from DNA from tissue samples from naturally infected animals and the genomes of the corresponding cultured isolates.

Supplementary Table 1 SureSelect target enrichment designed custom capture library RNA baits summary.

Supplementary Table 2 SNP analysis of *M. bovis* genomes sequenced directly form DNA samples spiked with BCG DNA at 10^–1^ and 10^–2^ with other *M. bovis* BCG genomes from the NVSL *M. bovis* genomes database.

Supplementary Table 3 Sequencing result of DNA from tissue samples spiked with serially diluted *M. bovis* BCG DNA at different concentration range without target enrichment.

Supplementary Table 4 SNP analysis of *M. bovis* genomes sequenced from tissue samples from experimentally infected animals with other *M. bovis* genomes from Mbovis-07B1 group from the NVSL *M. bovis* genomes database.

Supplementary Table 5 SNP analysis of *M. bovis* genomes sequenced directly form tissue sample with other *M. bovis* genomes in Mbovis-06A group.

Supplementary Table 6 SNP analysis of *M. bovis* genomes sequenced directly form tissue sample with other *M. bovis* genomes in Mbovis-07B group.

Supplementary Table 7 SNP analysis of *M. bovis* genomes sequenced directly form tissue sample with other *M. bovis* genomes in Mbovis-16C group.

Supplementary Table 8 SNP analysis of *M. bovis* genomes sequenced directly form tissue sample with other *M. bovis* genomes in Mbovis-17B1 group.

Supplementary Table 9 SNP analysis of *M. bovis* genomes sequenced directly form tissue sample with other *M. bovis* genomes in Mbovis-24 group.

Supplementary Table 10 SNP analysis of *M. bovis* genomes sequenced directly from DNA from tissue samples from naturally infected animals and the genomes of corresponding cultured isolates.
