# Supplementary figures and images for: Whole genome sequencing of Mycobacterium bovis directly from clinical tissue samples without culture
Source: Front Microbiol. 2023 May 18;14:1141651. doi: 10.3389/fmicb.2023.1141651 (PMC10232834; doi:10.3389/fmicb.2023.1141651)

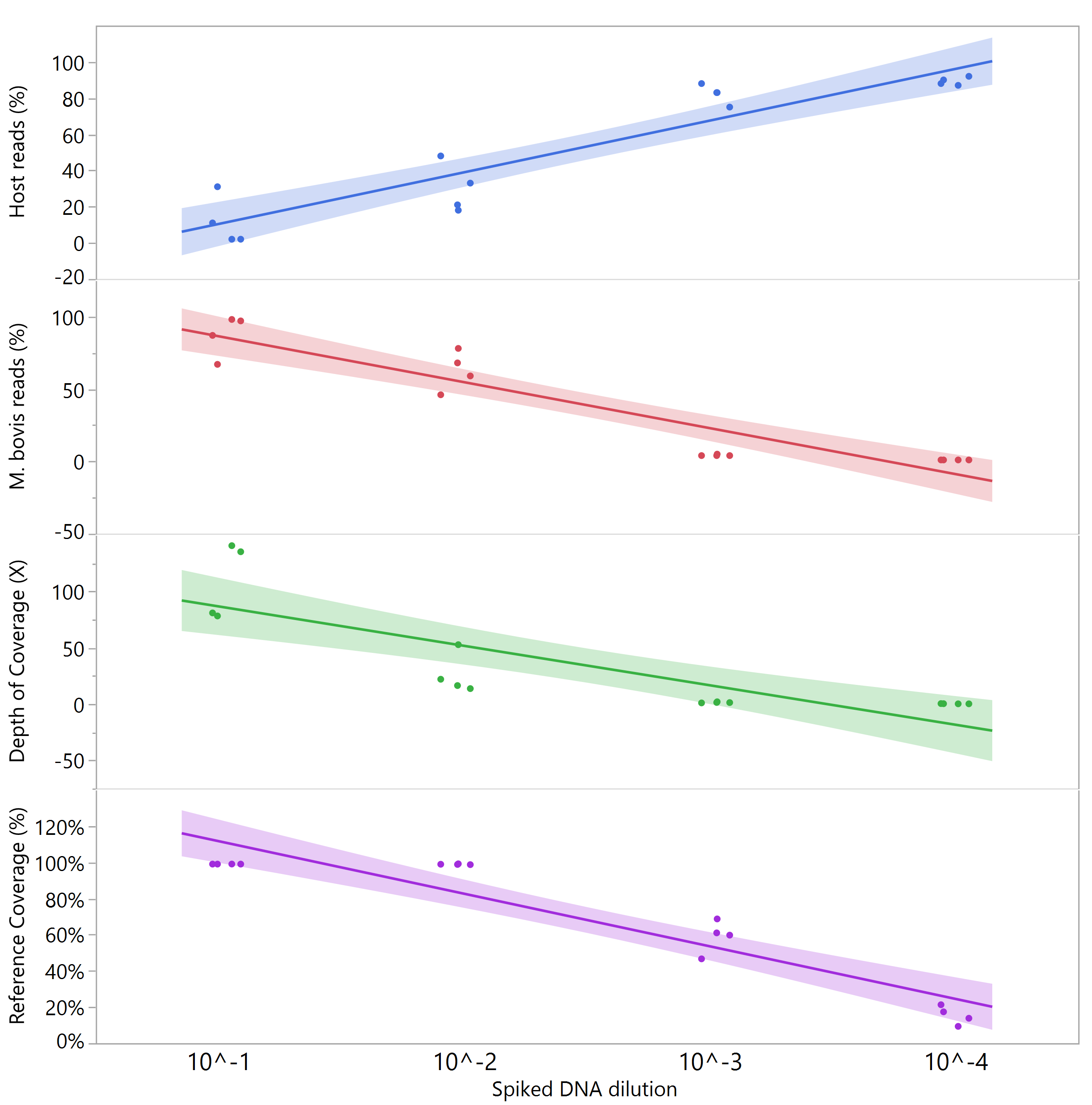

Supplement: Supplementary Figure 1 — Comparison of the percentages of reference genomes coverage, depth of coverage, M. bovis reads, host reads on the y axis by spiked DNA concertation on the x axis for each individual sample. Line of fit shows a linear regression with confidence intervals. [file Image_1.PNG]

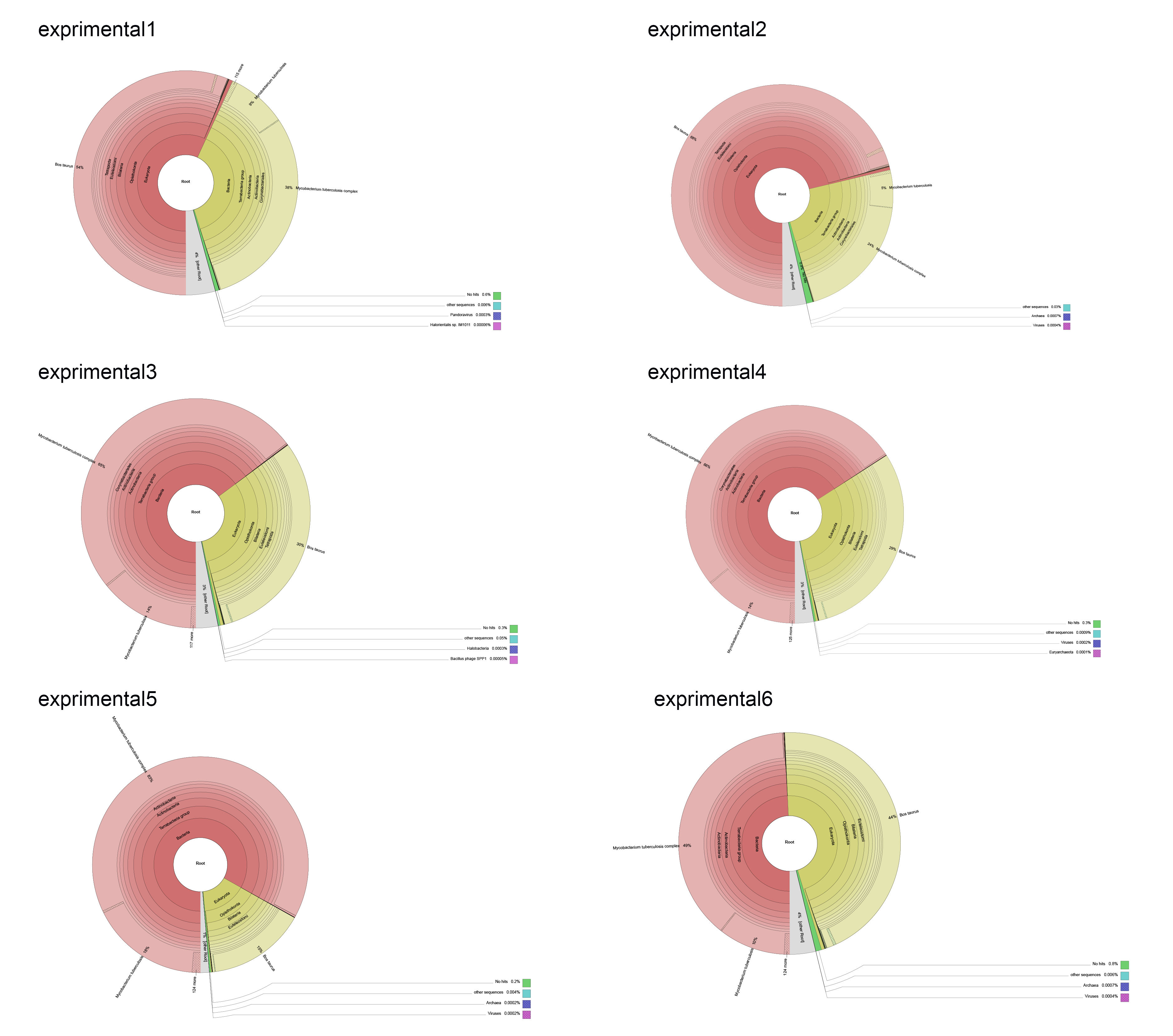

Supplement: Supplementary Figure 2 — Krona charts showing Kraken sequence reads classification and distribution from genomes sequenced directly from tissue samples from experimentally infected animals. Krona plots display the relative abundance and separate taxonomic domains using a radial space-filling display. Taxonomic domains are colored and arranged from the top level of the hierarchy at the center and progressing outward. [file Image_2.PNG]

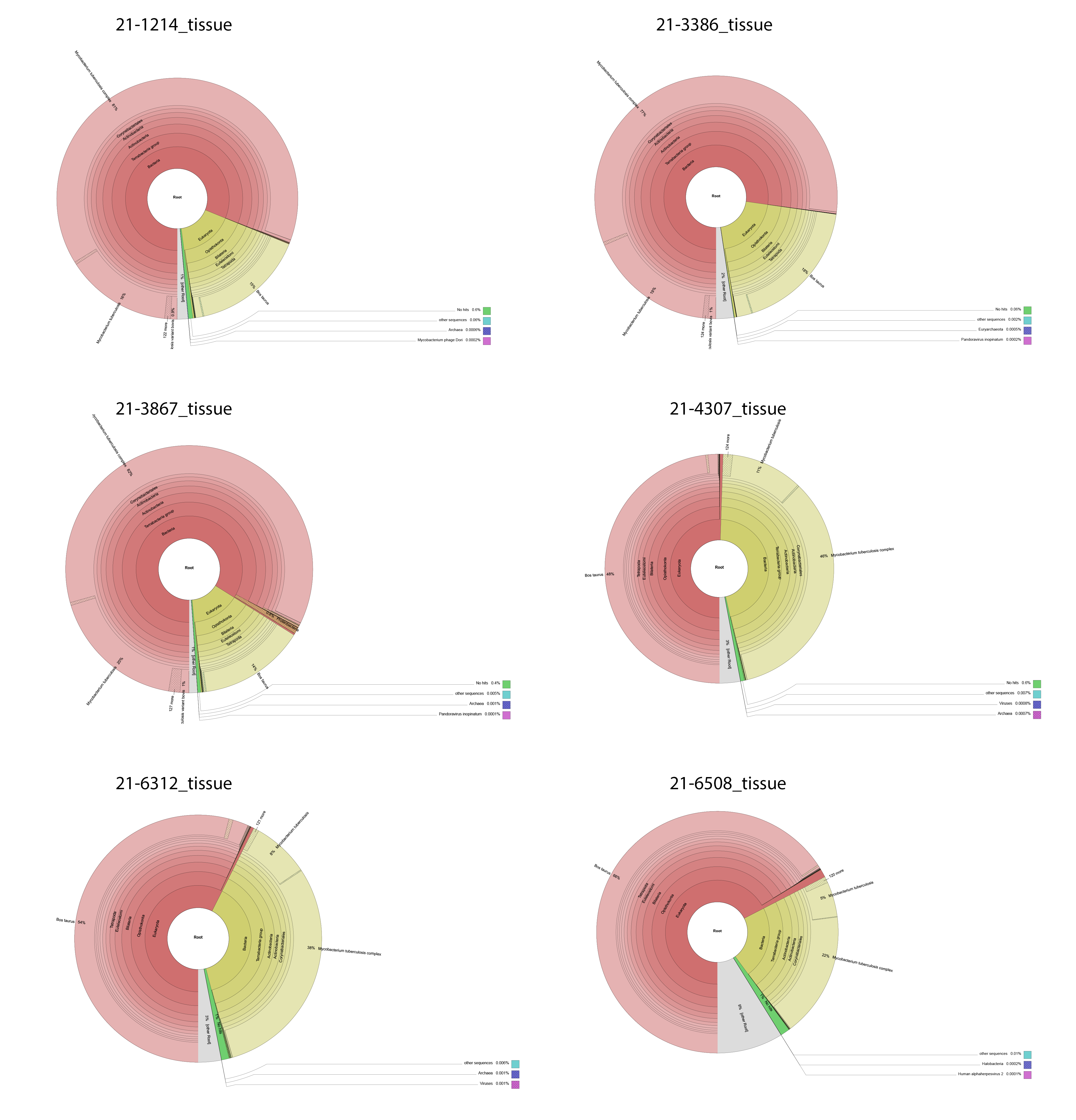

Supplement: Supplementary Figure 3 — Krona charts showing Kraken sequence reads classification and distribution from genomes sequenced directly from tissue samples from naturally infected animals. Krona plots display the relative abundance and separate taxonomic domains using a radial space-filling display. Taxonomic domains are colored and arranged from the top level of the hierarchy at the center and progressing outward. [file Image_3.PNG]

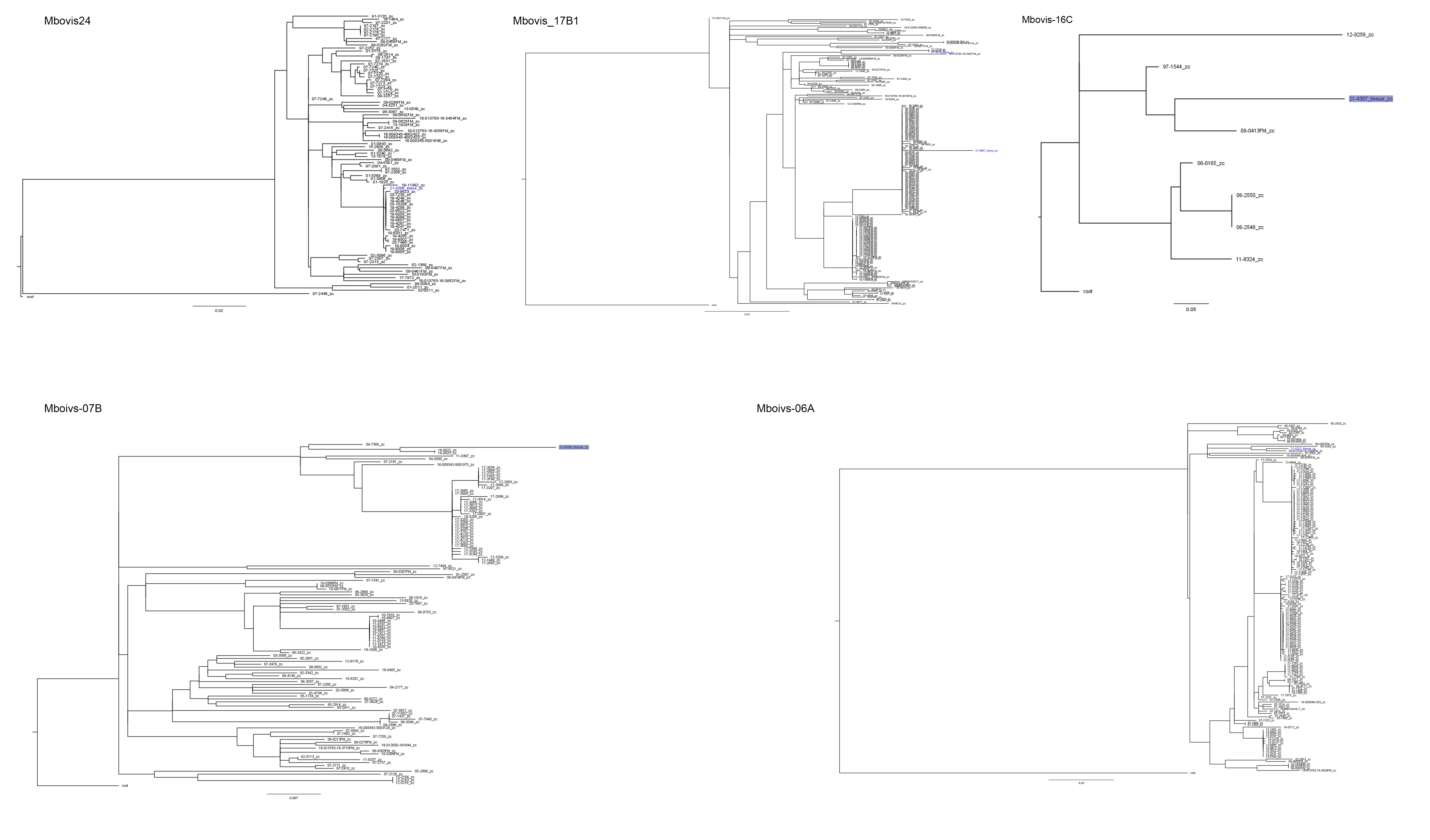

Supplement: Supplementary Figure 4 — Maximum-likelihood phylogenetic tree of the M. bovis genomes sequenced directly from DNA from tissue samples from naturally infected animals with other M. bovis genomes from NVSL genomes database. [file Image_4.PNG]

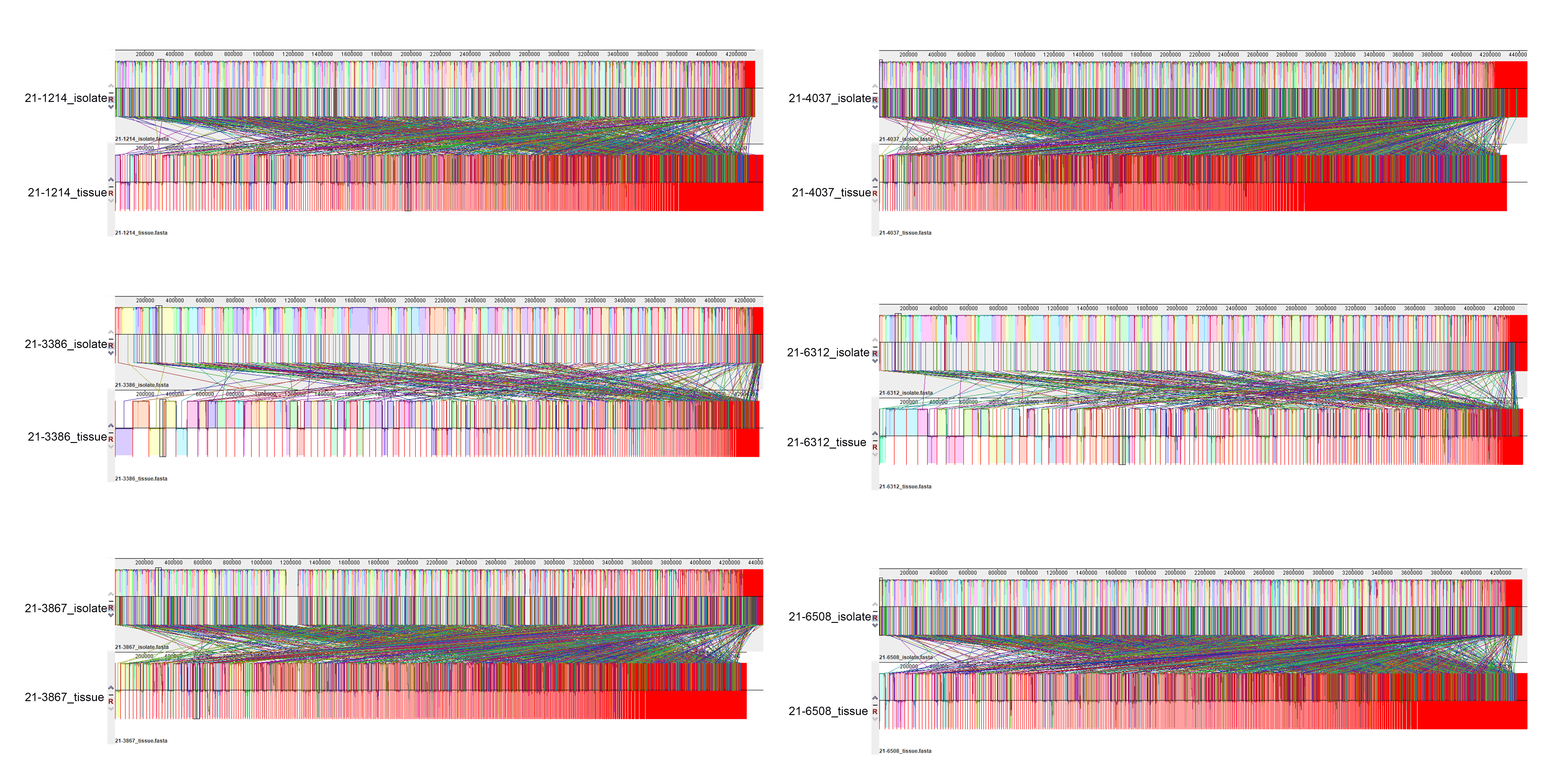

Supplement: Supplementary Figure 5 — Mauve multiple genome alignments of the M. bovis genomes sequenced directly from DNA from tissue samples from naturally infected animals and the genomes of the corresponding cultured isolates. [file Image_5.JPEG]
