# Supplementary material for: Whole genome sequencing of Mycobacterium bovis directly from clinical tissue samples without culture
Source: Front Microbiol. 2023 May 18;14:1141651. doi: 10.3389/fmicb.2023.1141651 (PMC10232834; doi:10.3389/fmicb.2023.1141651)
Supplement: Supplementary Table 3 — Sequencing result of DNA from tissue samples spiked with serially diluted M. bovis BCG DNA at different concentration range without target enrichment. [file Table_3.DOCX]

**Table S3.** Sequencing result of DNA from tissue samples spiked with serially diluted *M. bovis* BCG DNA at different concentration range without target enrichment.

| **Sample ID** | **BCG DNA dilution** | **Ct value** | **Mean read quality** | **Total reads** | **All mapped reads** | **Unmapped reads** | **Reference with coverage** | **Average depth of coverage** | **Quality SNP count** | **Group Placements** |
| --- | --- | --- | --- | --- | --- | --- | --- | --- | --- | --- |
| Sample1 | 10^^-1^ | 21.1 | 35.8 | 5,612,642 | 3,025 | 5,608,838 | 0.81% | 0.0X | 2 | No defining SNPs |
| Sample2 | 10^^-2^ | 23.4 | 35.6 | 7,250,658 | 54 | 7,250,600 | 0.09% | 0.0X | 0 | No defining SNPs |
| Sample3 | 10^^-3^ | 28.9 | 35.7 | 8,255,438 | 12 | 8,255,426 | 0.03% | 0.0X | 0 | No defining SNPs |
| Sample4 | 10^^-4^ | 30.3 | 35.6 | 7,278,504 | 10 | 7,278,488 | 0.01% | 0.0X | 0 | No defining SNPs |
